# Engaging Users on a Q&A Social Media Platform: The Influence of Disease Attributes and Message Features on Public Discussions of Depression

**DOI:** 10.3389/fpsyg.2021.712346

**Published:** 2021-09-24

**Authors:** Shuya Pan, Nan Yu, Yao Huang, Di Zhang

**Affiliations:** ^1^School of Journalism and Communication, Renmin University of China, Beijing, China; ^2^Nicholson School of Communication and Media, University of Central Florida, Orlando, FL, United States; ^3^Domestic News Department, Xinhua News Agency, Beijing, China

**Keywords:** Q&A social media, depression, message features, user engagement, the common sense model of illness self-regulation

## Introduction

Depression has affected over 54 million people in China and 322 million people around the world, which made it a great public health concern (WHO, 2017). Meanwhile, the public increasingly uses social media to discuss depression-related topics (Guntuku et al., 2017). In recent times, most studies regarding the engagement of users in depression-related discussions on social media had targeted popular platforms, such as Facebook, Twitter, Instagram, YouTube, or Sina Weibo (Cavazos-Rehg et al., 2016; Reece and Danforth, 2017; Pan et al., 2018). Research already indicated that people tend to show different behavioral patterns on different social media platforms (Alhabash and Ma, 2017; Waterloo et al., 2018). However, little research has been done to investigate how depression topics are discussed in a question-and-answer (Q&A) social media platform, where in-depth discussions of knowledge are encouraged.

To study this topic, we collected data from a popular Chinese Q&A platform Zhihu (in Chinese 知乎). Compared with other types of social media, Zhihu offers more than the conventional ways for users to engage in conversations in the Q&A threads. On Zhihu, once a question is proposed, other users can *view, follow*, or *answer* a question. This is defined as the Question-level (Q-level) of user engagement in this study. For each answer underneath each question, other users can *agree* or provide additional *comments*. This is defined as Answer-level (A-level) user engagement.

Scholars have attempted to identify the different meanings of such user engagement on social media. Based on two theoretical models, e.g., the heuristic-systematic model and the elaboration likelihood model, the action of “like” or “favorite” represents the peripheral route (heuristic) engagement which mostly involves emotional responses to the messages, whereas the action of “commenting” represents the central route (systematic) processing that involves more in-depth cognitive deliberation of messages (Liu et al., 2017). Following the previous literature, *answering* represents deeper cognitive engagement compared to *viewing* or *following* at the Q-level. While at the A-level, *agreeing* somehow is similar to the actions of *like* or *favorite*, while c*ommenting* indicates a deeper elaboration of the information.

In this study, we are specifically interested in how disease attributes and persuasive features of depression-related discussions on Zhihu are associated with these different levels of user engagement in both Q&A levels. We applied two sets of frameworks to identify the features of depression-related messages namely, The Common Sense Model of Illness Self-Regulation (CSM), to identify the disease attributes of depression being discussed on Zhihu, and the persuasion-related theories to identify the message characteristics that are persuasive or engaging.

The Common Sense Model describes how people make sense of certain illnesses or health threats and how representations of an illness from common sense would guide their coping strategies and efforts (Leventhal et al., 2003). The Common Sense Model (CSM) identifies five core attributes of illness that people commonly used to construct the representations of an illness, including *identity, cause, timeline, consequences*, and *controllability* (Please see Appendix A for detailed definitions of these categories). Research has shown that the different beliefs of people regarding illness representations can influence their emotional and behavioral responses. For example, perceptions of the timeline and consequences of diabetes were shown to affect the emotional distress of people, and control beliefs were found to be linked with treatment adherence (Paddison et al., 2010; Awasthi and Mishra, 2011). Rather than from the individual perspective, this research aimed to expand the CSM literature by analyzing Zhihu messages from the CSM framework and exploring how engaging are the messages that cover different disease attributes of depression on Zhihu.

The line of persuasion research also identified some message features that may elicit different responses to the messages (Dillard and Pfau, 2002). The message features considered in this study are the typical persuasive elements being identified, including narratives, affect, social support, imagery, source anonymity, and the crowdsourcing status (Please see Appendix A for a detailed definition of these concepts). First, previous research generally indicated that narratives are more engaging by immersing people into the stories (Clementson, 2020); Secondly, in terms of affect, the findings seem to indicate that negative messages are more sensational on social media (Jenders et al., 2013); Thirdly, a study found that supportive messages could create the in-group dynamics among social media users, which can trigger more commenting (Rus and Cameron, 2016); Fourthly, *imagery* messages are often processed faster and evoked emotional reactions on social media, such as “like” or “favorite” (Magnan and Cameron, 2015); Fifth, the anonymity of users can sometimes lead to higher engagement on social media when discussing sensitive topics (Suler, 2004), but anonymity was also found to be harmful in some online interactions for the lack of social cues (Laffey et al., 2006); Lastly, Zhihu enables certain level of crowdsourcing by encouraging other users to edit listed questions in order to come up with better and clearer ones. We would make the first-step exploration about the relationship between the crowdsourcing status and user engagement. In general, the present study plans to make unique theoretical contributions to the literature of persuasion by examining how Q&As with persuasive characteristics would have their specific ways of engaging users on Zhihu.

## Methods

### Sampling

After the Institutional Review Board (IRB) approval from a northern Chinese university, we used web-scraped data from the Zhihu for this study. A similar procedure has been used in the study of Pan et al. (2017). In specific, we first wrote the web crawler in computer programming language Python (Python Software Foundation in Delaware) to extract all questions with the keyword depression (in Chinese 抑郁症) during the time parameter ranged from November 1, 2017 to January 31, 2018. This time frame was chosen because Zhihu only allowed the program to extract the most recent 3-month when the study was conducted. We finally obtained a sample of 119 questions in total. At the Q-level, the following data were crawled from Zhihu: the ID information of senders if applicable, the posting date and time, the text of questions, the number of times each question is being edited, and the number of views, follows, and answers of all 119 questions.

In the second step, we identified the 20 most popular questions based on the ranking system of Zhihu out of the 119 questions. We wrote another web crawler in Python to extract all the answers to these 20 questions. Besides the text of the answers, the following data of answers were also collected: the posting date and time, the URL of images if applicable, and the number of agreements and comments of answers. There were altogether 29,157 answers provided to the 20 questions. We did a random selection of 40 answers from each of the 20 questions and constructed a sample of 800 answers.

The units of analysis for this study were per question and per answer. The coding schemes of the Q-level entries and A-level entries were detailed below.

### Coding Scheme

#### The Q-Level Coding

Message features considered at the Q-level include five disease attributes defined in CSM, whether applying narrative, source anonymity, and crowdsourcing status.

More specifically, the disease attributes defined in CSM were coded as: *identity* = 1, *cause* = 2, *timeline* = 3, *consequence* = 4, *controllability* = 5, and *others* = 6. *Narrative* was coded into two categories which were narrative = 1 and non-narrative = 0. *Anonymity* was coded into two categories which were anonymous = 1 and non-anonymous = 0. *Crowdsourcing* was coded based on the editing functions that Zhihu provides. Zhihu welcomes users to edit the questions to make them clearer to the audience. There were three areas in a question entry that can be edited which were (1) the question tags, (2) the question itself, and (3) the explanation of the question. The index of crowdsourcing was coded by adding up the numbers of the editing times in these three parts.

#### The A-Level Coding

At the A-level, we included the following message features: the CSM disease attributes defined in CSM, whether applying narrative, whether indicating social support, imagery, and the message affect.

Different from questions, answers are lengthier. Each answer normally covers the information of more than one category defined in CSM. Therefore, during A-level coding, we treated each category of CSM as a dichotomous variable, with the presence of each category, i.e., identity, cause, timeline, consequence, and controllability, coded as 1 and the non-presence coded as 0. In addition, *Narrative* was coded into two categories which were narrative = 1 and non-narrative = 0. *Affect* was coded into three categories which were positive = 1, negative = 2, and neutral = 0. *Social support* was coded into two categories which were yes = 1 and no = 0. *Imagery* was coded into two categories which were yes = 1 and no = 0.

The detailed definition of these categories is shown in Appendix A.

### Dependent Variables

User engagement for the Q-Level was measured by the *numbers of views, followers*, and *answers* to each question. User engagement for A-Level was measured by the *number of agreements and comments* to each answer.

### Coding Procedure

To establish inter-coder reliability, two graduate students whose first language is Chinese coded all 119 questions and 20% of 800 answers. The coders first went through the training session and discussed the meaning of each category. After the training session, two coders first rated all questions based on the coding scheme, and the disagreement was resolved by discussion. For the coding of 800 questions, both coders first rated 160 questions from 4 questions to test the reliability of the coding scheme and reached an agreement through discussion. The inter-coder reliability (Cohen's Kappa) ranges from 0.84 to 0.97 for all coded variables. After that, they independently coded half of the test sample.

## Data Analyses

The data analyses were conducted with STATA version 15 (developed by Stata Corp LLC in Taxes). We first performed data cleaning and preliminary analysis, yielding descriptive statistics and inter-rater reliability of each variable. A set of negative binomial regression analyses was conducted to examine how the proposed factors were associated with user engagement. This type of analysis was chosen because all dependent variables examined at the Q&A level were not normally distributed. In addition, considering the nested nature of answers, multi-level negative binomial regressions were conducted to examine the relationships between message features and user engagement at the A-level. Questions were treated as clusters to account for the non-independence of answers nested within the 20 questions. The significance level was set at *p* < 0.05.

### Descriptive Analyses

We first conducted the descriptive analyses of message features and user engagement of depression-related discussions on Zhihu. The detailed results are presented in Table 1.

**Table 1:** Descriptive statistics for the message features of Zhihu questions and answers.

	**Questions (*****N*** **= 119)**				**Answers (*****N*** **= 800)**			
	**Frequency**	**Percentage**	***M***	***SD***	**Frequency**	**Percentage**	***M***	***SD***
CSM								
Identity	24	20.17			294	36.75		
Cause	12	10.08			107	13.38		
Timeline	2	1.68			176	22		
Consequence	27	22.69			179	22.38		
Controllability	26	21.85			238	29.75		
Others	28	23.53						
Narrative	30	25.21			581	72.63		
Anonymity	41	34.45						
Crowdsourcing								
Tags			4.18	3.5				
Question			1.39	0.91				
Explanation			1.37	3.13				
Affect								
Positive					94	11.75		
Negative					118	14.75		
Neutral					588	73.5		
Social support					113	14.13		
**Imagery**					116	14.5		
Q-Number of followers		2,792.84	6,418.01					
Q-Number of views		803,872.14	2,127,582.9					
Q-Number of answers		367.13	853.1					
A-Number of agreements						227.78	947.71	
A-Number of comments						60.38	183.22	

*CSM, Common Sense Model of Illness Self-Regulation*.

At the Q-level, about 76.5% of the questions asking about illness attributes of depression mentioned by CSM, with consequence-related questions appeared 22.7% of the time, followed controllability (21.9%), identity (20.2%), cause (10.1%), and timeline (1.7%). The percentage of questions not belonging to CSM categories was about 23.5%. Meanwhile, most questions were not narrative questions, but rather asking basic facts and understanding of depression (74.8%). Finally, most questions were asked in a non-anonymous manner (65.6%).

At the A-level, identity appeared 36.8% of the time, followed by controllability (29.8%), consequence (22.4%), timeline (22.0%), and cause (13.4%). In terms of persuasive features, most answers used the narrative style (72.6%), showed no obvious affect (73.5%), and contained no supportive messages (85.9%) and no imagery (85.50%).

### Regression Analyses

In this study, we also conducted a set of negative binomial regressions to explore how disease attributes and message features were associated with user engagement in both Q&A levels (see Table 2).

**Table 2:** Negative binomial regression on user engagement in questions and answers as functions of message features.

**Independent Variables**	**Questions (*****N*** **= 119)**						**Answers (*****N*** **= 800)**			
	**Views**		**Follows**		**Answers**		**Agreements**		**Comments**	
	**B**	**IRR^a^**	**B**	**IRR**	**B**	**IRR**	**B**	**IRR^a^**	**B**	**IRR**
CSM										
Identity	0.52	1.67	0.58	1.79	0.23	1.26	0.69^***^	2.00	0.55^***^	1.72
Cause	0.16	1.18	−0.01	0.99	0.05	1.05	0.28	1.33	0.35	1.42
Timeline	2.76^*^	15.81	1.20	3.32	1.80	6.06	−0.36^*^	0.69	0.06	1.07
Consequence	−0.19	0.83	−0.18	0.83	0.00	1.00	0.68^***^	2.00	0.57^***^	1.76
Controllability	−0.16	0.85	−0.48	0.62	−0.36	0.70	0.11	2.00	0.15	1.16
Others	x	x	x	x	x	x				
Narrative (Yes)	−2.35^***^	0.10	−2.46^***^	0.09	−1.61^***^	0.20	−0.35^*^	0.70	−0.07	0.93
Anonymity (Yes)	0.13	1.14	0.40	1.49	−0.07	0.93				
Crowdsourcing	1.66^***^	5.23	1.79^***^	6.00	1.45^***^	4.28				
Affect										
Positive							0.91^***^	2.48	0.88^***^	2.41
Negative							0.30	1.35	0.56^**^	1.76
Neutral							x	x	x	x
Social support (Yes)							0.42^*^	1.51	0.05	1.06
Imagery (Yes)							0.24	1.27	0.47^*^	1.06
Constant	4.06^***^	0.27	58	2.53^***^	0.25	12.59	4.06^***^	58	2.53^***^	12.59

* *p < 0.05*,

** *p < 0.01, and*

*** *p < 0.001*.

a *The Incidence Rate Ratio (IRR), a measure of effect size, is calculated by exponentiating the regression coefficient*.

*CSM, Common Sense Model of Illness Self Regulation*.

#### CSM Disease Attributes and User Engagement

At the Q-level, generally, we found no significant difference in user engagement in five disease attributes of depression (identity, cause, timeline, consequence, and controllability). We only found that the timeline-related questions were more likely to increase views compared to questions not related to CSM disease attributes. At the A-level, the topics relating to depression identity or consequence significantly increased user engagement by receiving more agreements and comments.

#### Persuasive Message Features and User Engagement

At the Q-level, we examined whether the persuasive features *narrative, anonymity*, and *crowdsourcing* would affect user engagement in depression-related questions on Zhihu. The analyses indicated that narratively phrased questions were less likely to be viewed and followed by users. We also found that if a question was edited more times by users, it would be more likely to be viewed, followed, and answered.

At the A-level, we examined how *narrative, affect, social support*, and *imagery* presented in answers had affected user engagement. The analyses indicated that narratively phrased answers were less likely to be agreed upon. When the positive effect was mainly shown in answers, they were more likely to receive agreements and comments than the ones with neutral or negative affect. Answers with social support information were more likely to be agreed upon, and the answers with images were more likely to be commented on.

## Conclusion and Implications

Guided by the CSM and persuasion research framework, the data collected on a Q&A social media platform provided several interesting empirical findings regarding the relationships between message features and user engagement.

Our first theoretical contribution was to expand the application of CSM to online data by examining how users of a Q&A platform are engaged in the discussions of the attributes of the disease of depression which was identified by the CSM framework. For instance, this study found that discussions of depression identity or consequences can trigger more engagement among Zhihu users.

The second theoretical contribution was that we enriched the literature of persuasion by examining how some persuasive features in Q&A messages can engage users in different ways. We identified some surprisingly interesting patterns.

First, although previous research generally indicated that narratively phrased messages can be more engaging, our research showed the opposite. Users were more involved in the discussions of threads not related to personal experiences and feelings on Zhihu. As Zhihu continues to brand itself as a platform for knowledge sharing of objective or scientific information, the engagement of users might be influenced by such well-claimed behavioral norms on Zhihu, which leads them to be more involved in messages that tend to be factual or non-personal.

The study also revealed that messages with positive affect were mostly engaging by receiving more agreements and comments compared to messages with neutral or negative affect. It indicated that positive messages could arouse both emotional and cognitive responses among users. This finding is consistent with previous studies showing that people tend to use positivity to stay optimistic about a disease that seems very desperate for many people (Cameron and Jago, 2008), and the positive affect shown in depression-related messages was more engaging.

Our study also found that answers including supportive messages usually offered treatment suggestions or doctor recommendations to the seekers. Some also offered encouragement and emotional comfort. The presence of supportive messages received significantly more agreements but not more comments. Because Zhihu is not viewed as a dominantly support-exchange platform, supportive messages could hardly lead to more in-depth involvement such as giving comments.

Crowdsourcing, i.e., times of the questions being edited, also positively predicted user engagement. The more times a question being edited, the more likely that question is viewed, followed, or answered. Our study suggested that the function of crowdsourcing provided by a Q&A social media platform helps engage users. By viewing the editing process, users can possibly sense the importance of a question and pay more attention to it, which may motivate more engagement.

Finally, as Zhihu becomes the important channel for the Chinese public to discuss depression-related health topics, our data could also provide valuable knowledge by revealing the specific patterns of user engagement of this topic on Zhihu. Future depression-related interventions applied on Zhihu could apply such knowledge and prepare messages to engage users on this platform.

The current dataset has several limitations. First, we collected a sample from November of 2017 to January of 2018, the most recent sample that was available to us. Our data did not allow us to study a longitudinal change and limited the capability to compare the presence of different message features over time. Second, our data focused on one popular Q&A social media platform (Zhihu) in China. Similar platforms can also be explored such as Quora in the United States. Cross-country comparison is meaningful to understand how users from different countries discussed a particular health issue. Third, research has shown that people tend to react differently when facing different health conditions and behaviors (Shen et al., 2015). Therefore, the current data might only be applied to the discussions of depression. Despite all these, it is noteworthy to test whether such findings are also effective in explaining other health conditions.

## Data Availability Statement

The raw data supporting the conclusions of this article will be made available by the authors, without undue reservation.

## Ethics Statement

The studies involving human participants were reviewed and approved by Runze Wang, Institutional Review Board, School of Journalism and Communication, Renmin University of China. Written informed consent for participation was not required for this study in accordance with the national legislation and the institutional requirements.

## Author Contributions

SP was responsible for deciding the coding framework, data collection, analyses, and writing results. NY was responsible for conceptualization, writing introduction, methods and conclusion. YH was responsible for data cleaning and coding. DZ was responsible for conceptualization, editing, and finalizing the paper. All authors contributed to the article and approved the submitted version.

## Funding

This paper was supported by Journalism and Marxism Research Center, Renmin University of China (Project Number: 19MXG06).

## Conflict of Interest

The authors declare that the research was conducted in the absence of any commercial or financial relationships that could be construed as a potential conflict of interest.

## Publisher's Note

All claims expressed in this article are solely those of the authors and do not necessarily represent those of their affiliated organizations, or those of the publisher, the editors and the reviewers. Any product that may be evaluated in this article, or claim that may be made by its manufacturer, is not guaranteed or endorsed by the publisher.

## Appendix

**Appendix A:** Coding framework for message features of Zhihu QandA on depression.

**Category**	**Definition**
**CSM**	
Identity	Messages mentioning the illness label and associated symptoms
Cause	Messages mentioning the perceived cause of the condition
Timeline	Messages mentioning the predictive belief about how long the condition might last
Consequences	Messages mentioning how they have been affected by the condition physically, socially, psychologically, and other potential ways
Controllability	Messages mentioning the treatment options and one's ability to influence illness progression
Others	Messages not covering the above five elements of CSM
**Narrative**	
Narrative	Messages mainly stating personal experiences and feelings, normally in the form of storytelling
Non-narrative	Messages not related to personal experiences and feelings, but rather stating facts or opinions from the non-personal perspective
**Affect**	
Positive	Messages mainly indicating hope, optimism, and positive attitudes while fighting depression
Negative	Messages mainly expressing sadness, fear, anxiety, hopelessness, and other negative emotions
Neutral	Messages that take relatively neutral tones
**Imagery**	Messages including photos, graphics, animations, and other visual forms
**Social support**	Messages giving advices and help or showing empathy and comfort to another user, rather than just discussing public knowledge on depression

*CSM, Common Sense Model of Illness Self-Regulation*.
